# Psychometric properties of instruments to measure parenting practices and children’s movement behaviors in low-income families from Brazil

**DOI:** 10.1186/s12874-021-01320-y

**Published:** 2021-06-24

**Authors:** Widjane Sheila Ferreira Goncalves, Rebecca Byrne, Pedro Israel Cabral de Lira, Marcelo Tavares Viana, Stewart G. Trost

**Affiliations:** 1 Centre for Children’s Health Research, School of Exercise and Nutrition Sciences, Queensland University of Technology, Brisbane, Australia; 2 Federal University of Pernambuco, Recife, PE Brazil; 3 Centre for Children’s Health Research (CCHR), Level 6, 62 Graham St, South Brisbane, QLD 4101 Australia

**Keywords:** Psychometric properties, Parenting, Physical activity, Screen time, Sleep

## Abstract

**Background:**

Childhood obesity has increased remarkably in low and middle-income (LMIC) countries. Movement behaviors (physical activity, screen time, and sleep) are crucial in the development of overweight and obesity in young children. Yet, few studies have investigated the relationship between children’s movement behaviors and parenting practices because validated measures for use among families from LMIC are lacking. This study evaluated the psychometric properties of previously validated measures of young children’s physical activity, screen time, and sleep and parenting practices, translated and culturally adapted to Brazilian families.

**Methods:**

A total of 78 parent-child dyads completed an interviewer-administered survey twice within 7 days. Child physical activity, sedentary time and sleep were concurrently measured using a wrist-worn accelerometer. Internal consistency and test-retest reliability was assessed using McDonald’s Omega and Intraclass Correlation Coefficients (ICC’s). Concurrent validity was evaluated by calculating Spearman correlations between parent reported child behaviors and accelerometer measured behaviors.

**Results:**

Seventeen of the 19 parenting practices scales exhibited acceptable internal consistency reliability (Ω ≥ 0.70). Test-retest reliability ICC’s were acceptable and ranged from 0.82 - 0.99. Parent reported child physical activity was positively correlated with objectively measured total movement (rho= 0.29 - 0.46, *p* < .05) and energetic play (rho= 0.29 – 0.40, *p* < .05). Parent reported child screen time was positively correlated with objectively measured sedentary time; (rho = 0.26, *p* < .05), and inversely correlated with total movement (rho = - 0.39 – - 0.41, *p* < .05) and energetic play (rho = - 0.37 – - 0.41, *p* < .05). Parent reported night-time sleep duration was significantly correlated with accelerometer measured sleep duration on weekdays (rho = 0.29, *p* < .05), but not weekends.

**Conclusions:**

Measurement tools to assess children’s movement behaviors and parenting practices, translated and culturally adapted for use in Brazilian families, exhibited acceptable evidence of concurrent validity, internal consistency, and test-retest reliability.

**Supplementary Information:**

The online version contains supplementary material available at 10.1186/s12874-021-01320-y.

## Background

Childhood obesity in children under five is a global public health problem [1], and particularly problematic for children residing in low- and middle-income countries (LMIC) [2]. Worldwide, approximately three quarters of all overweight and obese children live in LMIC’s - with the highest prevalence in South America [2]. In North-east Brazil, it is estimated that 1 in 3 children are either overweight or obese [3]. Of concern, childhood obesity is associated with immediate and long-term health problems. The immediate problems of obesity in childhood include, elevated blood pressure, insulin resistance, and psychosocial health outcomes, such as lower self-esteem and higher prevalence of bullying [4–6]. The long-term health problems include increased risk of cardiovascular disease and type 2 diabetes [7]. Overweight and obesity during early childhood also increases the risk of obesity in adolescence and adulthood [8]. Children who are overweight in the first 5 years of life are five times more likely to be overweight at age 12 compared to children in the healthy weight range [9].

Movement behaviors are crucial in the prevention of overweight and obesity [10], with regular physical activity, limited sedentary screen time, and adequate sleep duration as the ideal combination [11]. The World Health Organization recommends that preschool-aged children spend a minimum of 3 hours a day in a variety of physical activities, with 60 minutes of moderate to vigorous physical activity (MVPA); accumulate less than 60 minutes of sedentary screen time; and sleep between 10 to 13 hours every day [12]. However, little is known about the proportion of South American children meeting these recommendations, including children from Brazil [13]. Currently there is substantial inconsistency in how obesity-related movement behaviors are measured, and validated assessment tools culturally adapted to LMIC families are lacking.

Parents plays a significant role in the development of children’s physical activity, screen time and sleep behaviors [14–17]. Parenting practices are the strategies parents use to assist or support children in their socialization goals, including the establishment of healthy lifestyle behaviors [18]. Logistic support, modeling, co-participation, and setting rules and limits are key parenting practices associated with healthful movement behaviors in young children [19–21]. The vast majority of studies examining parenting practices and child health behaviors, however, have been conducted in families from high-income countries [22, 23]. No studies have investigated parenting practices related to young children’s movement behaviours in families residing in LMIC countries. Accordingly, there is an urgent need for valid and reliable measures of parenting practices related to children’s movement behaviors for use in LMIC families, as currently no such measures exist. Knowing the parenting practices that support healthful movement behaviors in young children is a necessary prerequisite for the development of effective family-based interventions to prevent childhood obesity. To address these gaps in knowledge, the current study evaluated the internal consistency reliability, test-retest reliability and concurrent validity of previously validated measures of preschool-aged children’s physical activity, screen time, and sleep and parenting practices, translated and culturally adapted to Brazilian families.

## Methods

### Sample and recruitment

Parent-child dyads attending two Early Childhood Education and Care Centers (ECEC) from Caruaru, Brazil (1 rural; 1 urban) were invited to participate in the study. Prior to conducting the study, the Director from each Centre was contacted by the principal investigator to explain the research and obtain permission for the Centre to participate. Subsequently, a flyer was distributed to all parents of children between the ages three and six, inviting them to attend a meeting to explain the research project in detail. During this meeting, participant information sheets and consent forms were distributed. Parents who agreed to participate returned a signed informed consent form to the principal investigator. For parents with low literacy level and their children informed consent was obtained from a legally authorized representative. Participating parents were asked to nominate a day and time during the following week they could attend the center for data collection. All recruitment and data collection activities were completed between May and June 2019. The research was approved by the Human Research Ethics Committee of the Queensland University of Technology, Brisbane/Australia (Approval No. 1800001141), and the Department of Education of Caruaru, Brazil (Approval Letter March 1, 2019). All methods were carried out in accordance with relevant guidelines and regulations of the Human Research Ethics Committee at Queensland University of Technology.

### Protocol

Parents completed a survey measuring parenting practices and children’s movement behaviors translated into Portuguese and adapted for use in Brazilian families [24]. The Portuguese and English versions of the survey are included as Supplementary material. Depending on literacy level, parents could complete the survey themselves, or have it administered to them as an interview. Each interview took approximately 45 minutes to complete. Parents with multiple children enrolled in the ECEC were instructed to complete the survey in relation to their first-born.

After completing the survey, participating children were outfitted with an accelerometer-based motion sensor on the non-dominant wrist to be worn 24 hours/day (with the exception of bathing or swimming) for seven consecutive days. On completion of the 7-day monitoring period, parents returned the accelerometer to a member of the research team at the ECEC and completed the parent survey a second time. Participating parent-child dyads received an USD $3.65 gift (soccer ball, skipping rope or peteca) in return for their time and effort.

### Measures

The following socio-demographic information were collected: child’s sex, date of birth, ethnicity, attendance at ECEC (part-time vs full-time), caregivers’ age and gender, level of education, marital status, current employment status, household income, financial support from government ‘Bolsa Família Programme’ and number of residents at home.

#### Parenting practices

Parenting practices related to physical activity and screen time were measured using translated and culturally adapted versions of the measurement scales developed by Vaughn [24, 25]. These scales measure parenting practices used to control or support children’s physical activity and screen time. Controlling parenting practices included rules around active play indoors (12 items), rules around active play outdoors (4 items), use of physical activity to reward/control behaviour (5 items), limiting outdoor play due to weather (2 items), limiting, or monitoring of screen time (8 items), and use of screen time to reward/control child behaviour (4 items). Supportive parenting practices included explicit modelling and enjoyment of physical activity (10 items), verbal encouragement for physical activity (6 items), logistic support for sports (3 items), logistic support for active play (3 items), importance and value of physical activity (3 items), support/reinforcement from other adults (3 items), exposure to TV (3 items), and explicit modelling and enjoyment of screen time (6 items) [25]. Parenting practices related to sleep were measured using items adapted from the Bedtime Routines Questionnaire [26]. This questionnaire consists of 5 scales measuring routine behavior (4 items), routine environment (4 items), reactivity (4 items), adaptive activities (7 items), and maladaptive activities (6 items).

#### Parent reported child health behaviours

Children’s physical activity was measured using a translated version of the Burdette outdoor playtime recall [27]. Parents reported the amount of time their child spent playing outdoor considering a typical weekday and a typical weekend day in the last month.

Child screen time was assessed using an adaptation of an instrument used in the Australian InFANT study [28]. Parents reported their child’s screen time on a normal weekday and a normal weekend day based on a number of electronic devices. This included watching television programs and DVDs and videos viewing, using a computer, playing with an electronic game system (e.g. Nintendo DS, Playstation, Xbox), and using smartphones, iPads or Tablets. Night-time sleep duration was measured using items adapted from the Prevention of Overweight in Infancy randomized control trial [29]. Parents reported the time their child usually went to bed at night and the time the child woke up in the morning to start the day. These items assessed sleep on weekdays and weekend days separately.

### Accelerometer-measured movement behaviors

Daily time spent in physically active movement behaviors was measured using the ActiGraph GT3X+ accelerometer (ActiGraph Corporation, Pensacola FL, USA). Raw accelerometer data (30 Hz) was downloaded and processed into physical activity metrics using a random forest physical activity classification algorithm specifically developed for children under five [30]. This validated machine learning algorithm uses 20 features extracted from the raw tri-axial acceleration signal to classify activity type and quantify daily time spent in sedentary activities (sitting or lying down), light-intensity activities and games (slow walking, standing, standing arts and crafts), walking, running, and moderate-to-vigorous intensity activities and games (active games with balls, riding bikes/scooters). In a free-living evaluation, the random forest algorithm exhibited an overall classification accuracy of greater than 80%. Total moment was calculated by summing daily time spent in light-intensity activities and games, walking, running, and moderate-to-vigorous activities and games; while energetic play was calculated by summing daily time spent in walking, running, and moderate-to-vigorous activities and games. Nighttime sleep duration was measured using the sleep/wake detection algorithm developed by Van Hees et al. [31]. Non-wear periods were identified by summing the 15 second windows in which the standard deviation of the vector magnitude was < 13 mg for >= 30 consecutive minutes [32]. The child’s accelerometer data was included in the analyses if they had ≥ 5 days in which wear time was 10 hours (600 minutes) or longer.

### Statistical analysis

Means and standard deviations were calculated for the parenting practices scales and parent reported movement behaviors. The internal consistency of scales was evaluated using McDonald’s Omega. Omega is a preferable indicator of internal consistency reliability because the measurement model assumed by Cronbach’s alpha (equal factor loadings or essential tau equivalency) is typically not tenable in applied research [33]. One-week test-retest reliability was assessed by calculating Intraclass Correlation Coefficients (ICC); mode: two-way mixed; type: agreement). Omega was considered acceptable at ≥ 0.70, while ICC’s were considered acceptable at ≥ 0.75 [34]. Evidence of concurrent validity for parent reported child physical activity, screen time, and sleep were evaluated by calculating Spearman correlations with accelerometer measured activity metrics, including sedentary time, total movement, energetic play, and sleep duration. Omega statistics were calculated using the MBESS package in R [35]. All other statistical procedures were performed using SPSS statistical software version 25. Significance was set at an alpha level of 0.05.

## Results

Of the 132 families attending the two childcare services, 78 parent child-dyads (38 from an urban ECEC service and 40 from a rural ECEC service) consented to participate. Children were a mean age of 4.6 ± 0.8 years, 53% male, 42% mixed race, 52% attended the rural ECEC service, and 71% attended childcare half-time. Descriptive data regarding the parents is presented in Table 1. Due to low literacy, most parents (*N* = 70, 90 %) completed the survey as an interview-administered survey.

**Table 1:** Parent demographics and descriptive characteristics

Variables	Parents
	N (%)
**Sex**	
Female caregiver	71 (92)
**Age** (years)	
≤24	11 (14)
Between 25-35	43 (56)
> 36	23 (30)
**Marital status**	
Single	17 (22)
Married	19 (25)
Living with partner	34 (44)
Separated/divorced	6 (8)
Widowed	1 (1)
**Employment status**	
Employed full-time	42 (55)
Employed part-time	8 (10)
Casually employed	13 (17)
Unemployed or retired	14 (18)
**Household income**^a^	
<= 1 wage	53 (69)
Between 1 and 2 wage	20 (26)
> 2 wage	4 (5)
**Level of education**	
No study	3 (4)
Elementary school	42 (54)
High school	19 (25)
Tertiary education	10 (13)
Post-graduation	3 (4)
**Number of residents**	
≤ 4	51 (66)
> 4	26 (34)
**‘Bolsa família’ programme**^b^	
Yes	53 (69)

^a^ 1 wage was equivalent to R$997 monthly in Brazilian Real in 2019 (equivalent 190 USD)

^b^ government assistance program for low-income families

Internal consistencies (Omega), test-retest reliability coefficients (ICC) and means ± SD for the parenting practices scales are reported in Table 2. McDonald’s Omega for 17 of the 19 of the parenting practices scales were acceptable. Low internal consistencies were observed for the “support/reinforcement from other adults” (Ω = 0.46 – 0.50) and “maladaptive activities on sleep” (Ω = 35 – 0.38) scales. ICC’s for the parenting practices scales were acceptable and ranged from ICC = 0.82 - 0.99. However, the “logistic support for sports” scale exhibited low test-retest reliability (ICC = 0.47). Means and SD’s for parenting practice constructs measured at Time 1 and Time 2 were almost identical.

**Table 2:** Internal consistencies, test-retest reliability coefficients, and means (±SD) for the parenting practices scales

Scale (#items)	Internal consistency (Ω)		Test-retest reliability (ICC)	Mean ± SD	
	Time 1	Time 2		Time 1	Time 2
**Controlling physical activity parenting practices**					
Rules around active play indoors	0.85	0.81	0.90	2.3 ± 0.5	2.2 ± 0.4
Rules around active play outdoors	0.71	0.79	0.96	3.2 ± 1.4	3.2 ± 1.4
Use of PA to reward/control child behavior	0.88	0.88	0.92	2.9 ± 1.4	3.0 ± 1.3
Limiting outdoor play due to weather	0.83	0.80	0.90	3.8 ± 1.7	3.7 ± 1.5
Limiting or monitoring of screen time	0.85	0.89	0.97	1.8 ± 0.9	1.8 ± 0.9
Use of screen time to reward/control child behavior	0.86	0.89	0.95	2.7 ± 1.5	2.8 ± 1.5
**Supportive physical activity parenting practices**					
Explicit modeling and enjoyment of PA	0.78	0.76	0.95	2.9 ± 0.8	2.9 ± 0.7
Verbal encouragement for PA	0.71	0.71	0.84	3.1 ± 1.0	3.0 ± 1.0
Logistic support for sports	0.70	0.63	0.47	0.8 ± 0.4	1.3 ± 0.5
Logistic support for active play	0.80	0.71	0.91	2.6 ± 1.5	2.6 ± 1.3
Importance and value of PA	0.83	0.78	0.82	4.2 ± 0.7	4.0 ± 0.7
Support/reinforcement from other adults	0.50	0.46	0.75	3.8 ± 0.7	3.7 ± 0.7
**Supportive screen parenting practices**					
Exposure to ST	0.75	0.77	0.99	4.3 ± 2.3	4.3 ± 2.3
Explicit modeling and enjoyment of ST	0.79	0.76	0.82	3.7 ± 1.1	3.7 ± 0.9
**Sleep parenting practices**					
Consistency: routine behavior	0.79	0.82	0.86	4.0 ± 0.9	4.1 ± 0.9
Consistency: routine environment	0.78	0.75	0.88	4.3 ± 0.8	4.2 ± 0.7
Reactivity	0.68	0.70	0.87	1.4 ± 0.6	1.5 ± 0.6
Adaptive activities	0.73	0.77	0.91	3.7 ± 0.8	3.6 ± 0.7
Maladaptive activities	0.35	0.38	0.76	2.5 ± 0.6	2.5 ± 0.6

*Legend*: *Ω* McDonald’s Omega, *ICC* Intraclass correlation coefficient, *PA* Physical activity, *SD* Standard deviation, *ST* Screen time

Means and standard deviations for the parent reported and accelerometer measured children’s movement behaviors are reported in Table 3. Averaged over weekdays and weekend days, parent reported children’s physical activity ranged from 192 to 195 minutes per day; screen time ranged from 197 to 199 minutes per day, while sleep duration ranged from 606 to 607 minutes per day. Based on the accelerometer data, children, on average, accumulated 437 minutes per day of sedentary time, 377 minutes per day of total movement, and 33 minutes per day of active play. Average night-time sleep duration was 505 minutes per day.

**Table 3:** Descriptive data for the parent reported and accelerometer measured children’s movement behaviors

		Mean ± SD		
		Week days	Weekend days	Average of weekdays and weekend days
**Parent reported (minutes)**				
*Physical activity*	*Time 1*	153 ± 71	236 ± 118	195 ± 88
	*Time 2*	146 ± 63	236 ± 112	192 ± 82
*Screen time*	*Time 1*	184 ± 105	210 ± 144	197 ± 117
	*Time 2*	175 ± 99	222 ± 142	199 ± 112
*Sleep*	*Time 1*	584 ± 71	629 ± 76	607 ± 59
	*Time 2*	585 ± 70	626 ± 72	606 ± 61
**Accelerometer measured (minutes)**				
*Energetic play*		31 ± 15	42 ± 26	33 ± 16
*Total movement*		361 ± 72	446 ± 106	377 ± 80
*Sedentary time*		437 ± 89	448 ± 101	437 ± 86
*Sleep duration*		507 ± 69	509 ± 93	505 ± 63

Test-retest reliability coefficients and 95% confidence intervals for the parent reported children’s movement behaviors are displayed in Table 4. ICC’s for children’s movement behaviors on weekdays, weekend days and the average of weekend and weekdays were excellent and ranged from 0.85 to 0.97.

**Table 4:** Test-retest intra-class correlation coefficients and 95% confidence intervals for the parent reported children’s movement behaviors

	Weekdays ICC (95% CI)	Weekend days ICC (95% CI)	Average day ICC (95% CI)
**Physical activity**	0.92 (0.87, 0.94)	0.97 (0.95, 0.98)	0.96 (0.95, 0.98)
**Screen time**	0.85 (0.77, 0.90)	0.93 (0.89, 0.95)	0.94 (0.90, 0.96)
**Sleep**	0.94 (0.90, 0.96)	0.90 (0.84, 0.93)	0.93 (0.90, 0.96)

*Legend*: *CI* Confidence intervals, *ICC* Intraclass correlation coefficient

Spearman correlations between parent reported physical activity, screen time and sleep, and accelerometer measured physical activity, sedentary time and sleep are reported in Table 5. On weekdays, weekends, and the average of weekdays and weekends, parent reported physical activity was positively and significantly correlated with total movement (rho= 0.29 - 0.46, *p* < .05) and active play (rho=0.29 – 0.40, *p* < .05). Averaged over weekdays and weekend days, parent reported screen time was significantly and positively correlated with objectively measured sedentary time; (rho = 0.26, *p* < .05), and significantly inversely correlated with total movement (rho = - 0.39 – - 0.41, *p* < .05) and active play (rho = - 0.37 – - 0.41, *p* < .05). Parent reported night-time sleep duration was significantly and positively correlated with accelerometer measured sleep duration on weekdays (rho = 0.29, *p* < .05), but not weekends.

**Table 5:** Spearman correlations between parent report measures of child physical activity, screen time and sleep, and accelerometer measured children’s movement behaviors

		Weekdays (Spearman rho)			
		Sedentary time	Energetic play	Total movement	Sleep
Physical activity	T1	**-0.37**	**0.39**	**0.46**	0.14
	T2	**-0.33**	**0.40**	**0.40**	0.14
Screen time	T1	0.21	**-0.27**	**-0.32**	-0.12
	T2	**0.26**	**-0.42**	**-0.40**	-0.15
Sleep	T1	**-0.30**	0.19	0.19	**0.29**
	T2	-0.20	0.16	0.13	0.22
		Weekend Days (Spearman rho)			
Physical activity	T1	**-0.23**	**0.29**	**0.29**	0.07
	T2	**-0.23**	**0.30**	**0.33**	0.07
Screen time	T1	**0.25**	**-0.29**	**-0.29**	0.04
	T2	0.20	**-0.26**	**-0.25**	-0.01
Sleep	T1	0.13	0.06	0.10	-0.01
	T2	0.12	0.02	0.14	-0.05
		Average of Weekday and Weekend Day (Spearman rho)			
Physical activity	T1	**-0.31**	**0.39**	**0.44**	0.05
	T2	**-0.30**	**0.39**	**0.41**	0.07
Screen time	T1	**0.26**	**-0.37**	**-0.39**	-0.11
	T2	**0.24**	**-0.41**	**-0.41**	-0.14
Sleep	T1	-0.11	0.18	0.16	0.15
	T2	-0.03	0.11	0.12	0.07

*Legend*: In bold type = *p* ≤ 0.05; T1 = Time 1; T2 = Time 2

## Discussion

This study evaluated the psychometric properties of instruments to measure children’s movement behaviors and parenting practices in low-income families from Brazil. To our knowledge, it is the first study evaluating tools to measure these concepts in disadvantaged families from urban and rural Brazil and more broadly, families residing in LMIC. The internal consistency for 17 of the 19 parenting practices scales were acceptable, and with the exception of “logistic support for sports”, test-retest reliability for the parenting practices scales and children’s movement behaviors were high (≥ 0.75). Correlations between parent report measures of child physical activity, screen time and sleep, and accelerometer measured children’s movement behaviors were statistically significant and in expected direction. These findings indicate that the measures have evidence of concurrent validity, internal consistency, and test-retest reliability in Brazilian families.

The parenting practices scales exhibited satisfactory internal consistency reliability at both Time 1 and Time 2. However, there were some notable exceptions. The “support/reinforcement from other adults” and “maladaptive activities on sleep” scales exhibited low internal consistency. Comparing our results to those reported in the validation study involving families in the United States [25], it is interesting to note that a low alpha coefficient (α = 0.54) was also reported for the “support/reinforcement from other adults” scale. This result could be explained, at least in part, by the disparate content of the items. One of the items asked parents to rate their confidence to get their child to be physically active at home and did not refer to other adults in the child’s life. Another item asked about other adults in their child’s life enforcing rules about screen time - a completely different movement behavior from physical activity. In relation to the poor internal consistency of the “maladaptive activities on sleep” scale, the original validation study reported evidence that the items functioned as a unidimensional scale; nevertheless, it presented lower internal consistency (α = 0.69) than the other bedtime routine scales (α = 0.74 - 0.90) [26]. In the current study, the low internal consistency was predominantly driven by the responses to the items about ‘watching TV’ and ‘playing video games’ in the hour before going to bed, which exhibited negative factor loadings. This suggests that these two items were not assessing the same underlying construct and that, within our sample, the frequency of screen-based activities prior to bedtime was unrelated to the frequency of other activities thought to stimulate children before bed and delay sleep onset and quality (e.g., playing active games and watching television). However, when these two items were deleted, Omega only increased to 0.58 – 0.59, which is still below acceptable.

Overall, ICCs for the parenting practices scales and the parent reported child behaviors were high. Only the scale “logistic support for sports” did not demonstrate acceptable test-retest reliability. The low correlation observed between repeat assessments of this scale is likely a function of floor effects and restriction of range. In contrast to many high-income countries, there is no provision of organized sport or physical activity programs for Brazilian children under the age of five [36]; and there are no opportunities for parents to watch their child during training sessions or competitions. Furthermore, families in this study were from low-income communities, thus paying fees for the child to have sports lessons, classes, or play organised sports was not relevant.

Parent reported child physical activity was positively correlated with objectively measured total movement and energetic play, while inversely correlated with screen time. Interestingly, the validity coefficients observed in the present study were considerably larger in magnitude than those reported by Burdette et al. [27] for children in the United States (*r* = 0.20). The discrepancy in findings may be attributable, at least in part, to differences in the accelerometer data processing methods. Burdette et al [27] used the average vector magnitude recorded by the RT3 tri-axial accelerometer as a criterion measure of physical activity which serves as an indicator of the total volume of physical activity and not the type and intensity of physical activity in which children were participating. In the current study, a validated, state-of-the-art machine learning physical activity classification model for free living pre-schoolers was applied to the accelerometer data to derive physical activity metrics that captured both overall movement and time in energetic play [30]. Therefore, the criterion physical activity measure used in the current study may have had less random measurement error, resulting in stronger associations between self-reported and device-based measures of children’s physical activity behaviour.

As evidence of concurrent validity, parent reported child screen time was positively correlated with objectively measured sedentary time, and inversely correlated with total movement and energetic play. However, the amount of child screen time reported by parents warrants discussion. On average, parents reported their child as engaged in screen time for three hours per day (175 – 184 minutes) on weekdays and just over three and a half hours per day (210 – 222 minutes) on weekend days. Only 14% of children in this sample met the WHO sedentary screen time recommendation of less than 1 hour/day [11]. These findings are consistent with other studies conducted in preschool-aged children from South America. In a study of 302 Brazilian children under six, de Carvalho Cremm et al. [37] reported that 61% of children watched television for more than 2 hours daily. A cross-sectional study investigating 45 preschool- aged children from Chile, indicated that 100% of children spent more than 2 hours in front of television daily [38]. Finally, an Argentinian study of 183 children aged 5 years and younger reported that 40% of children spent more than 2 hours on screen time daily [39]. Therefore, our results, supported by other findings from studies conducted in other LMIC, suggests that the majority of young children in South America are not meeting the global recommendation for daily screen time. This finding underscores the urgent need for the development of effective parenting interventions to reduce screen time in preschool-aged children in South American countries, and indeed in similar LMICs.

Parent reported night-time sleep duration was significantly correlated with accelerometer measured sleep duration on weekdays, but not weekends. This finding could be a result of parents being more able to recall sleep duration on weekdays because children follow a routine during the week. This contrasts with weekend nights, when there may be no routine in place and parents have difficulty recalling their child’s bedtime and wake up time. In addition, because of the seven-day monitoring protocol, some children (9%) did not have actigraphy-based estimates of sleep duration on both weekend nights. This may have negatively impacted the reliability of the calculation of weekend sleep duration.

Although significant positive correlations were observed between the two, there were substantial differences between the parent reported and accelerometer measured children’s movement behaviors. However, such differences are expected given that the self-report measures and device-based measures assessed different outcomes. Regarding physical activity behavior, the Burdette recall instrument asked parents to report outdoor playtime [27], while the data obtained from the accelerometer measured movement time regardless of the child’s physical location. Reporting time in outdoor play is therefore a slightly different construct to accelerometer measures of total movement and energetic play. When a child is outside it does not necessarily mean that the child is being active as they could be engaged in sedentary behaviors or intermittent light intensity activities requiring little movement. In relation to screen time, parents reported their child’s exposure to screen-based devices on weekdays and weekend days. However, the accelerometer measured total sedentary time across the day and did not consider exposure to screens. Screen time may not be entirely sedentary time, and not all sedentary time involves screens.

This study had several strengths. To our knowledge, this is the first study to evaluate the concurrent validity, internal consistency reliability, and test-retest reliability of measurement tools to assess parenting practices and children’s movement behaviors in a LMIC country, using measurement tools that have gone through a rigorous process of translation and cognitive testing prior to validation [24]. Second, this study recruited a diverse sample from urban and rural disadvantaged Brazilian families with low literacy levels. Lastly, concurrent validity was assessed using advanced machine learning accelerometer data processing methods, allowing for a detailed examination of children’s physical activity, sedentary behavior, and sleep duration. There were however several limitations. The results were obtained on a relatively small sample from only two communities of Brazil. Therefore, the results may not be generalizable to all communities in Brazil and other LMIC. Further studies are needed to evaluate the psychometric properties of these measurement tools in larger more diverse samples, and in other LMIC communities. Additionally, 91% of the participants completed the survey as an interview and the results may not be fully generalizable to situations where the instrument is completed as a self-administered questionnaire. However, the participants had low levels of formal education and lacked the literacy skills required to read and complete the measures on their own. It is very likely that future studies may have to use the same mode of administration if they wish to complete studies in families from rural areas in LMIC.

## Conclusions

In conclusion, previously validated measures of children’s movement behaviors and parenting practices, translated and culturally adapted for use in Brazilian families, exhibited strong evidence of concurrent validity, internal consistency reliability, and test-retest reliability. Considering these findings, the measures could be used in studies to examine the children’s compliance with 24-hour movement guidelines, and the relationships between parenting practices and children’s obesity-related behaviors in disadvantaged families from Brazil. Future studies should evaluate the psychometrics properties of these scales in families living in other LMIC’s who are also facing childhood obesity challenges.

## Supplementary Information

**Additional file 1.**

## Data Availability

The dataset supporting the conclusions of this article can be made available upon request after approval by the authors. Please direct inquiries to widjane.ferreiragoncalves@hdr.qut.edu.au.

## Abbreviations

BRQ: Bedtime routine questionnaire
ECEC: Early Childhood Education and Care
LMIC: Low-middle income countries
WHO: World Health Organization
