# Supplementary material for: Psychometric properties of instruments to measure parenting practices and children’s movement behaviors in low-income families from Brazil
Source: BMC Med Res Methodol. 2021 Jun 24;21:129. doi: 10.1186/s12874-021-01320-y (PMC8223314; doi:10.1186/s12874-021-01320-y)
Supplement: Supplementary file 1 — Additional file 1. [file 12874_2021_1320_MOESM1_ESM.pdf]

PSYCHOMETRIC PROPERTIES OF INSTRUMENTS TO MEASURE PARENTING PRACTICES AND CHILDREN'S MOVEMENT BEHAVIORS IN LOW-INCOME FAMILIES FROM BRAZIL

Widjane Sheila Ferreira Goncalves[1*]; Rebecca Byrne[2]; Pedro Israel Cabral de Lira[3]; Marcelo Tavares Viana[4]; Stewart G. Trost[5]

[1]Centre for Children's Health Research, School of Exercise and Nutrition Sciences, Queensland University of Technology, Brisbane, Australia

widjane.ferreiragoncalves@hdr.qut.edu.au

[2]Centre for Children's Health Research, School of Exercise and Nutrition Sciences, Queensland University of Technology, Brisbane, Australia.

ra.byrne@qut.edu.au

[3]Federal University of Pernambuco, Recife - PE, Brazil.

lirapic@ufpe.br

[4]Federal University of Pernambuco, Recife - PE, Brazil.

mtviana0@hotmail.com

[5]Centre for Children's Health Research, School of Exercise and Nutrition Sciences, Queensland University of Technology, Brisbane, Australia.

s.trost@qut.edu.au

Corresponding author: Professor Stewart Trost, Centre for Children's Health Research (CCHR), Level 6, 62 Graham St, South Brisbane, QLD, 4101, Australia.

s.trost@qut.edu.au

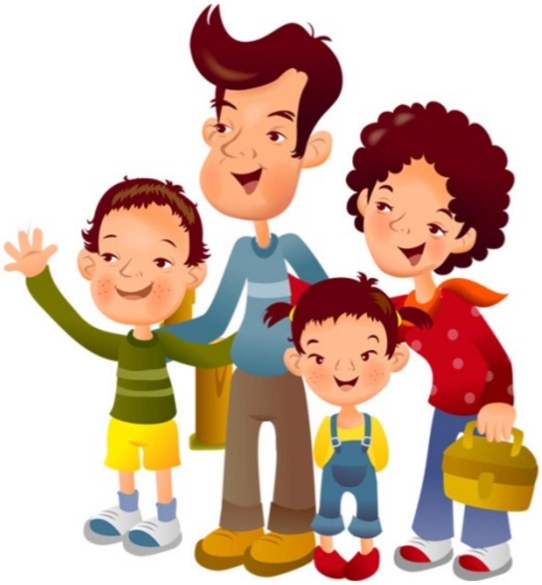

Family Health Survey

- The purpose of this survey is to obtain information about your health behavior and the health behaviors of your child.
- Please respond only about your oldest child aged 3 to 6 who is enrolled at Child Care/ Pre-school.
- Be as accurate as you can – there are no right or wrong answers.
- All information is strictly confidential.
- Please try not to miss any questions and provide only one answer for each question.
- If you are unsure on how to answer any question, please do not hesitate to ask the research team for help.
- Your responses are important to us.
- Answering this survey will take about 45 minutes of your time.

Identification

Child's Name: ____

Child Care/Pre-school: ______________________________________

Class: ( ) Berçario II ( ) Pré I ( ) Pré II

Child Care ID: __________ Participant ID: __________

Your Child's Personal Information

This section of the survey is about your child.

1. What is your child's sex?

☐ Female

☐ Male

2. What is your child's date of birth?

_______ / _______ / _______
day month year

3. What is your child ethinicity?

☐ Caucasian

☐ Mongoloid

☐ Mixed-race

☐ Afro Brazilians

☐ Indigenous

4. What time does your child attend a day care center/pre-school?

☐ Half-time (morning or afternoon)

☐ Full-time (morning and afternoon)

About You….

This section of the survey is about you and your family.

5. How old are you?

- ☐ Up to 24 years old
- ☐ Between 25 and 35 years old
- ☐ Between 36 and 45 years old
- ☐ Older than 46 years old

6. What is your relationship to the child?

- ☐ Female adult caregiver (e.g. mother, mother-in-law, grandmother, aunt)
- ☐ Male adult caregiver (e.g. father, father-in-law, grandfather, uncle)

7. What is your highest level of education?

- ☐ No study
- ☐ Incomplete Elementary School
- ☐ Complete Elementary School
- ☐ Incomplete High School
- ☐ Complete High School
- ☐ Incomplete Tertiary Education
- ☐ Complete Tertiary Education
- ☐ Post-graduation

8. What is your marital status?

- ☐ Single
- ☐ Married
- ☐ Living with partner
- ☐ Separated or divorced
- ☐ Widowed

9. How would you describe your current employment status?

☐ Employed full-time ‘full day’ (include self-employed)

☐ Employed part-time (include self-employed)

☐ Casually employed

☐ Unable to work due to health problems

☐ Unemployed

☐ Retired

10. What was the income range of the family in the last month?

☐ Up to R$998

☐ Between R$998 and R$1497

☐ Between R$1498 and R$1996

☐ Greater than R$1997

11. Do you receive financial support from the government through the ‘Bolsa Família Programme’ because your child attends the Child Care/ Pre-school?

☐ Yes

☐ No

12. How many people live in your house? (Including you).

☐ One

☐ Two

☐ Three

☐ Four

☐ Five or more

About your Child's Physical Activity

The following questions are about your child's weekly physical activity.

Think a moment about a normal weekday for your child in the last month.

13. How much time would you say your child spends playing outdoors on a normal **weekday**?

Hours: ☐☐ Minutes: ☐☐

Now think about a normal weekend day for your child in the last month.

14. How much time would you say your child spends playing outdoors on a normal **weekend day**?

Hours: ☐☐ Minutes: ☐☐

About your Child's Screen Time

The following questions are about your child's screen time. This includes the use of television, iPads/computer tablets, smartphones, game consoles and computers.

15. Thinking about the last month, how much time does your child spend doing each of the following activities at home? Please report separately for weekdays and weekend days.

	On a normal **weekday**<br>Monday - Friday<br>hours/minutes	On a normal **weekend day**<br>Saturday & Sunday<br>hours/minutes
**A. Watching TV/DVDs**	☐☐ **hrs** ☐☐ **mins**	☐☐ **hrs** ☐☐ **mins**
**B. Using a computer**	☐☐ **hrs** ☐☐ **mins**	☐☐ **hrs** ☐☐ **mins**
**C. Playing with a video game system (e.g. Nintendo DS, Playstation, Xbox, etc.)**	☐☐ **hrs** ☐☐ **mins**	☐☐ **hrs** ☐☐ **mins**
**D. Using smartphones and iPads/Tablet Computer**	☐☐ **hrs** ☐☐ **mins**	☐☐ **hrs** ☐☐ **mins**

About your Child's Sleep

The following questions are related to your child's sleeps routine.

Thinking about the last month…

16. What time does your child usually fall asleep at night?

On a normal **weekday**: Hours: ☐☐ Minutes: ☐☐

On a normal **weekend day**: Hours: ☐☐ Minutes: ☐☐

17. What time does your child usually wake up in the morning to start the day?

On a normal **weekday**: Hours: ☐☐ Minutes: ☐☐

On a normal **weekend day**: Hours: ☐☐ Minutes: ☐☐

About your Interactions with your Child in Relation to Physical Activity and Screen Time

The following questions are about the ways you interact with your child in relation to physical activity and screen time. We are interested in what **you** do and how **you** feel. Please read each question and select the best answer for you. Take your time and answer as accurately as possible.

18. How **often** is the TV in your house on when people are at home?

☐ Very Rarely

☐ Rarely

☐ Sometimes

☐ Often

☐ Very Often

☐ Always

19. Place an "X" in the box below that best represents how often your child is allowed to do each of the following activities while playing **inside your house.**

	Always	Sometimes	Never
a. hopping, skipping, or galloping	☐	☐	☐
b. running around	☐	☐	☐
c. chasing	☐	☐	☐
d. rough housing or wrestling	☐	☐	☐
e. jumping from a height	☐	☐	☐
f. flipping (somersault) or tumbling	☐	☐	☐
g. climbing	☐	☐	☐
h. swinging or hanging	☐	☐	☐
i. balancing	☐	☐	☐
j. piling up pillows and jumping on them	☐	☐	☐
k. throwing, kicking, or bouncing a ball	☐	☐	☐

20. Place an "X" in the box below that best represents how much you agree or disagree with each of the following statements.

	Strongly disagree	Disagree	Neither agree nor disagree	Agree	Strongly agree
a) When my child is **inside the house** their play should be calm and quiet.	☐	☐	☐	☐	☐
b) When **inside the house**, my child can use toys and equipment for physically active play (*for example, gross motor activities like running, jumping, hopping, or tumbling*).	☐	☐	☐	☐	☐

21. How **often** do you ask your child to calm down their **indoor play**?

☐ Never

☐ Very Rarely

☐ Rarely

☐ Sometimes

☐ Often

☐ Very Often

22. Place an "X" in the box below that best represents how often you do each of the following things related to your child's **outdoor play**.

**How often do you…**	**Never**	**Very Rarely**	**Rarely**	**Sometimes**	**Often**	**Very Often**
a. ask your child **not** to run when they are playing **outside?**	☐	☐	☐	☐	☐	☐
b. ask your child to try and stay clean when playing **outside**.	☐	☐	☐	☐	☐	☐
c. let your child play **outside** on hot days.	☐	☐	☐	☐	☐	☐
d. let your child play **outside** on cold days.	☐	☐	☐	☐	☐	☐
e. ask your child to calm down their **outdoor** play?	☐	☐	☐	☐	☐	☐
f. ask your child not to get their clothes dirty while he/she is playing **outside.**	☐	☐	☐	☐	☐	☐

23. Do you limit the amount of time your child watches TV, videos, or movies **during the week** (Monday – Friday)?

☐ Yes

☐ No ⟶ **If no, skip to question 25**

24. About how much time is your child allowed to watch **each weekday**?

Hours: ☐☐ Minutes: ☐☐

25. Do you limit the amount of time your child watches TV, videos, or movies **on the weekend** (Saturday - Sunday)?

☐ Yes

☐ No ⟶ **If no, skip to question 27**

26. About how much time is your child allowed to watch each **weekend day**?

Hours: ☐☐ Minutes: ☐☐

27. Do you limit the amount of time your child plays video games **during the week** (Monday - Friday)?

☐ Yes

☐ No ⟶ **If no, skip to question 29**

28. About how much time is your child allowed to play video games **each weekday** (Monday - Friday)?

Hours: ☐☐ Minutes: ☐☐

29. Do you limit the amount of time your child plays video games **on the weekend** (Saturday - Sunday)?

☐ Yes

☐ No ⟶ **If no, skip to question 31**

30. How much time is your child allowed to play video games **each weekend day?**

Hours: ☐☐ Minutes: ☐☐

31. Place an "X" in the box below that best represents how often you do each of the following things.

How often do you…	Never	Very rarely	Rarely	Some-times	Often	Very often
a. offer TV, video, or movie time to your child as a reward for good behavior?	☐	☐	☐	☐	☐	☐
b. take away TV, video, or movie time as a punishment for bad behavior?	☐	☐	☐	☐	☐	☐
c. offer sports or physical activities to your child as a reward for good behavior?	☐	☐	☐	☐	☐	☐
d. use sports or physical activities to get your child to do something? (for *example: "You can't go outside to play until you eat your lunch".*)	☐	☐	☐	☐	☐	☐.

32. Place an "X" in the box below that best represents how much you agree or disagree with each of the following statements.

**I tightly monitor the <u>time</u> my child…**	Strongly disagree	Disagree	Neither agree nor disagree	Agree	Strongly agree
a. watches TV or Videos during the **week** (Monday - Friday)	☐	☐	☐	☐	☐
b. watches TV or Videos on the **weekend** (Saturday - Sunday)	☐	☐	☐	☐	☐
c. plays video games during the **week** (Monday - Friday)	☐	☐	☐	☐	☐
d. plays video games on the **weekend** (Saturday - Sunday)	☐	☐	☐	☐	☐

33. How many **days per week** does your family have the television on during **breakfast**?

☐ 0 days

☐ 1 day

☐ 2 days

☐ 3 days

☐ 4 days

☐ 5 days

☐ 6 days

☐ 7 days

34. How many **days per week** does your family have the television on during **evening meal**?

☐ 0 days

☐ 1 day

☐ 2 days

☐ 3 days

☐ 4 days

☐ 5 days

☐ 6 days

☐ 7 days

35. Place an "X" in the box below that best represents how often you do each of the following things.

**How often…**	**Never**	**Very rarely**	**Rarely**	**Some-times**	**Often**	**Very often**
a. does **your child** get extra TV, video, or movie time as a reward?	☐	☐	☐	☐	☐	☐
b. does **your child** get extra outside time as a reward?	☐	☐	☐	☐	☐	☐
c. do **you** use TV time to control your child's behavior? (*for example: "If you don't stop that you will not be able to watch TV today."*)	☐	☐	☐	☐	☐	☐
d. do **you** use sports or physical activities to control your child's behavior? (*for example: "If you don't stop that you will not be able to go to soccer/dance class this afternoon."*)	☐	☐	☐	☐	☐	☐
e. do **you** take outside time away from your child for bad behavior?	☐	☐	☐	☐	☐	☐

36. Place an "X" in the box below that best represents how much you agree or disagree with each of the following statements.

		Strongly disagree	Disagree	Neither agree nor disagree	Agree	Strongly agree
a.	**I** have control over how much TV my child watches.	☐	☐	☐	☐	☐
b.	**Others adults** in my child's life make it hard to get my child to be physically active.	☐	☐	☐	☐	☐
c.	**My family** is physically active.	☐	☐	☐	☐	☐
d.	**I** enjoy watching TV/movies with my child.	☐	☐	☐	☐	☐

37. How much do **you** enjoy physical activity or sport?

☐ Don't enjoy

☐ Sort of enjoy

☐ Really enjoy

☐ Thoroughly enjoy

38. How much do **you** enjoy watching TV or movies during your free time?

☐ Don't enjoy

☐ Sort of enjoy

☐ Really enjoy

☐ Thoroughly enjoy

39. How often does **your family** use physical activities or sports as a form of family recreation? *(for example, going on bike rides together, hiking, and playing soccer/volleyball)*?

☐ Rarely

☐ Once in a while

☐ Relatively often

☐ Frequently

40. How often do **you** go to **your child's** sporting events, lessons, or other organized physical activities with them? *(for example, watch your child in a football or volleyball competition, perform in a dance recital, or training sessions)*?

- ☐ Rarely
- ☐ Once in a while
- ☐ Relatively often
- ☐ Frequently

41. How valuable is it to **you** that **your child** be physically active?

- ☐ Not valuable
- ☐ Of little value
- ☐ Moderately
- ☐ Valuable
- ☐ Very valuable

42. During the **past year** has an adult in your family **paid fees** so your child could take lessons, classes, or play sports involving moderate or vigorous physical activity? *(for example, dance, soccer, basketball, swimming, gymnastics)*?

- ☐ Yes
- ☐ No ⟶ **If no, skip to question 44**

43. For how many activities have you or other adults paid fees?

☐☐ **Activities**

44. How often do you use **your own behavior** to encourage your child to be physically active?

- ☐ I don't use my own behavior to encourage my child to be active.
- ☐ I rarely use my own behavior to encourage my child to be active.
- ☐ I often use my own behavior to encourage my child to be active.
- ☐ I always use my own behavior to encourage my child to be active.

45. How often do **you** enroll your child in sports?

- ☐ I rarely enroll my child in sports.
- ☐ I enroll my child once in a while.
- ☐ I frequently enroll my child in sports.
- ☐ I always enroll my child in sports.

46. During the **last month**, how many times have **you** taken your child to play at a park, playground or other leisure area of the region?

☐☐ **Time(s) in last month**

47. Place an “X in the box below that best represents how much you agree or disagree with each of the following statements.

		Strongly disagree	Disagree	Neither agree nor disagree	Agree	Strongly agree
a.	**I** am in charge of how much TV my child watches during their free time at home.	☐	☐	☐	☐	☐
b.	**I** can get my child to be physically active at home.	☐	☐	☐	☐	☐
c.	**Others adults** who have contact with my child make it hard to enforce household rules about how much time s/he is allowed to watch TV.	☐	☐	☐	☐	☐
d.	**I** like being physically active with my child.	☐	☐	☐	☐	☐

48. Place an "X" in the box below that best represents how often you do the following things during a normal week.

**During a normal week, how often...**		Never	Very rarely	Rarely	Some-times	Often	Very often
a.	do **you** tell your child how sedentary habits (*for example, sitting for a long time)* can be harmful to health?	☐	☐	☐	☐	☐	☐

b.	do **yo**u watch TV or videos with your child?	☐	☐	☐	☐	☐	☐
c.	do **you** send your child outside to play so you can do household chores in your home?	☐	☐	☐	☐	☐	☐
d.	do **you** take your child to the park to play?	☐	☐	☐	☐	☐	☐

49. Place an "X" in the box below that best represents how often the following things happen during a normal week.

**During a normal week, how often...**		**Never**	**Very rarely**	**Rarely**	**Some-times**	**Often**	**Very often**
a.	do **you** tell your child that physical activity is good for health?	☐	☐	☐	☐	☐	☐
b.	does **your** behavior encourage your child to be sedentary? (*for example, watching TV for a long time.*)	☐	☐	☐	☐	☐	☐
c.	do **you** praise your child for participating in sports or physical activities?	☐	☐	☐	☐	☐	☐
d.	do **you** turn on the TV, a video, or movie for your child when the weather is bad? (*for example, when it is raining, too hot or too cold.)*	☐	☐	☐	☐	☐	☐
e.	do **you** say things to encourage your child to do physical activities or play sports?	☐	☐	☐	☐	☐	☐

50. Place an "X" in the box below that best represents how often each of the following things happen during a normal week.

**During a normal week, how often...**		**Never**	**Very rarely**	**Rarely**	**Some-times**	**Often**	**Very often**
a.	does your child **hear you say** that you were too tired to do physical activities?	☐	☐	☐	☐	☐	☐
b.	does your child **see you** watching TV or movies?	☐	☐	☐	☐	☐	☐
c.	do **you** play sports, active games, or do other physical activities with your child?	☐	☐	☐	☐	☐	☐

d. do **you** try to get your child to play outside when the weather is nice?	☐	☐	☐	☐	☐	☐
e. do **you** transport your child to a place where they can be physically active or play sports?	☐	☐	☐	☐	☐	☐

51. Place an "X" in the box below that best represents how often each of the following things happen during a normal week.

**During a normal week, how often...**	**Never**	**Very rarely**	**Rarely**	**Some-times**	**Often**	**Very often**
a. does your child **hear you** talk about participating in a sport or being physically active?	☐	☐	☐	☐	☐	☐
b. does your child **see you** doing, or going to do, something that is physically active (*for example, walking, biking, playing sports*)?	☐	☐	☐	☐	☐	☐
c. do **you** turn on the TV, a video, or movie for your child so you can do household chores in your home?	☐	☐	☐	☐	☐	☐
d. do **you** try to get your child to be physically active instead of watching TV?	☐	☐	☐	☐	☐	☐
e. do **you** say things to encourage your child to spend less time being sedentary? (*for example, "Stop watching TV and go outside and play".)*	☐	☐	☐	☐	☐	☐

52. Place an "X" in the box below that best represents how important each of the following statements is to you.

**How important is it for you that your child…**	**Unimportant**	**Of little importance**	**moderately important**	**important**	**very important**
a. participate in sports?	☐	☐	☐	☐	☐
b. be physically active when they grow up?	☐	☐	☐	☐	☐

About your Interactions with your Child in Relation to Sleep

The following questions are about the ways you interact with your child in relation to sleep.

To answer the questions remember that:

Bedtime routines are a set sequence of activities that occur regularly in the same order and with the same caretaker before a child goes to bed. Place an "X" in the box that best represents your child's sleep routine in the last month.

**53. During *weeknights* for the past month, how often did your child…**		**Almost never**	**Sometimes**	**Half the time**	**Often**	**Nearly always**
a.	perform the **same activities** in the hour before going to bed *(for example, bathe, brush teeth, read/listen to story, listen to music)*?	☐	☐	☐	☐	☐
b.	perform activities **in the same order** before going to bed *(for example, bathe, brush teeth, read/listen to story, listen to music)*?	☐	☐	☐	☐	☐
c.	sleep **in the same place** *(for example, in his/her bed, in parent's bed, on couch)*?	☐	☐	☐	☐	☐
d.	go to bed **at the same time** (within 10 minutes of the scheduled time)?	☐	☐	☐	☐	☐
e.	get put to bed **by the same person**?	☐	☐	☐	☐	☐

**54. During *weekend nights* for the past month, how often did your child…**		**Almost never**	**Sometimes**	**Half the time**	**Often**	**Nearly always**
a.	perform the **same activities** in the hour before going to bed *(for example, bathe, brush teeth, read/listen to story, listen to music)*?	☐	☐	☐	☐	☐
b.	perform activities **in the same order** before going to bed *(for example, bathe, brush teeth, read/listen to story, listen to music)*?	☐	☐	☐	☐	☐
c.	sleep **in the same place** *(for example, in his/her bed, in parent's bed, on couch)*?	☐	☐	☐	☐	☐
d.	go to bed **at the same time** (within 10 minutes of the scheduled time)?	☐	☐	☐	☐	☐
e.	get put to bed **by the same person**?	☐	☐	☐	☐	☐

55. How upset does your child get if he or she does NOT…	Not at all	A little	Moderately	Quite a bit	Extremely
a. perform the **same activities** in the hour before going to bed *(for example, bathe, brush teeth, read/listen to story, listen to music)*?	☐	☐	☐	☐	☐
b. perform activities **in the same order** before going to bed (*for example, bathe, brush teeth, read/listen to story, listen to music*)?	☐	☐	☐	☐	☐
c. sleep **in the same place** *(for example, in his/her bed, in parent's bed, on couch*)?	☐	☐	☐	☐	☐
d. go to bed **at the same time** (within 10 minutes of the scheduled time)?	☐	☐	☐	☐	☐
e. get put to bed **by the same person**?	☐	☐	☐	☐	☐

56. In the past month, in the hour before going to bed, how often did your child…	Almost never	Sometimes	Half the time	Often	Nearly always
a. Read/listen to a story?	☐	☐	☐	☐	☐
b. Play with games or toys?	☐	☐	☐	☐	☐
c. Have active play (such as roughhouse or run around?	☐	☐	☐	☐	☐
d. Watch TV?	☐	☐	☐	☐	☐
e. Play video games?	☐	☐	☐	☐	☐
f. Listen to music?	☐	☐	☐	☐	☐
g. Have a snack or drink?	☐	☐	☐	☐	☐
h. Take a shower/bath?	☐	☐	☐	☐	☐
i. Brush teeth?	☐	☐	☐	☐	☐
j. Use the toilet?	☐	☐	☐	☐	☐
k. Hug/kiss caregiver (*for example, a good night kiss*)?	☐	☐	☐	☐	☐
l. Say goodnight to family members?	☐	☐	☐	☐	☐
m. Get tucked in?	☐	☐	☐	☐	☐

n.	Put on pajamas?	☐	☐	☐	☐	☐
o.	Say prayers?	☐	☐	☐	☐	☐
p.	Cuddle with caregiver? (*for example, the child sat on the lap of the caregiver and was hugged with him/her)*	☐	☐	☐	☐	☐

You’re finished!

Thank you for your time and effort!

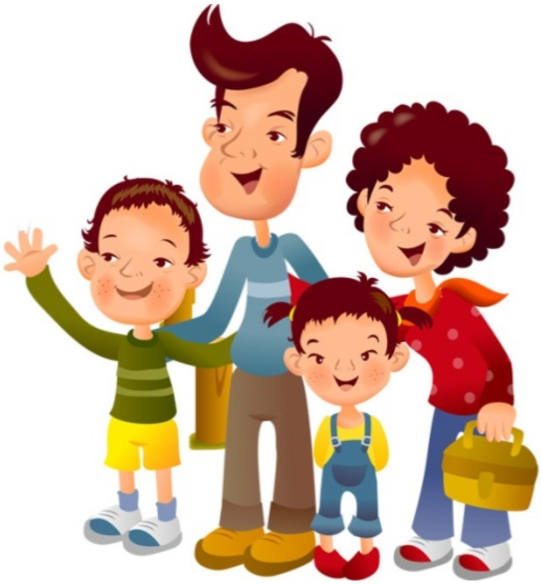

Questionário Saúde da Família

- O objetivo deste questionário é obter informações sobre o seu comportamento de saúde e o comportamento de saúde da sua criança.
- Por favor, responda as questões apenas sobre a sua criança mais velha entre 3 e 6 anos de idade que esteja matriculado na creche/pré-escola.
- Seja o mais preciso possível – não há respostas certas ou erradas.
- Todas as informações são rigososamente confidenciais.
- Por favor, tente responder a todas as perguntas e forneça apenas uma resposta para cada questão.
- Caso você não tenha certeza de como responder alguma questão, peça ajuda à equipe de pesquisa.
- Suas respostas são muito importantes para nós.
- Responder à este questionário tomará cerca de 45 minutos do seu tempo.

Identificação

Nome da Criança: ______________________________

Creche/Pré-escola: ______________________________

Série: ( ) Berçario II ( ) Pré I ( ) Pré II

Creche ID: __________ Participante ID: __________

Informações Pessoais da sua Criança

Esta parte do questionário é sobre a sua criança.

1. Qual é o sexo da sua criança?

☐ Feminino

☐ Masculino

2. Qual é a data de nascimento da sua criança?

_______ / _______ / _______
dia mês ano

3. Qual é a raça/cor da sua criança?

☐ Branco(a)

☐ Amarelo(a)

☐ Pardo(a)/mestiço(a)

☐ Preto(a): moreno(a)/negro(a)

☐ Indígena

4. Qual o período que a sua criança frequenta a creche/pré-escola?

☐ Meio período (manhã ou tarde)

☐ Período integral (manhã e tarde)

Sobre você...

Esta parte do questionário é sobre você e sua família.

5. Quantos anos você tem?

☐ Até 24 anos

☐ Entre 25 e 35 anos

☐ Entre 36 e 45 anos

☐ Mais do que 46 anos

6. Qual a sua relação com a criança?

☐ Adulto responsável do sexo feminino (ex.: mãe, madrasta, avó, tia)

☐ Adulto responsável do sexo masculino (ex.: pai, padrasto, avô, tio)

7. Qual é o seu nível mais alto de escolaridade?

☐ Não tenho estudos

☐ Ensino fundamental incompleto

☐ Ensino fundamental completo

☐ Ensino médio incompleto

☐ Ensino médio completo

☐ Ensino superior incompleto

☐ Ensino superior completo

☐ Pós-graduação

8. Qual é o seu estado civil?

☐ Solteiro(a)

☐ Casado(a)

☐ Morando com o companheiro(a)

☐ Separado(a) ou divorciado(a)

☐ Viúvo(a)

9. Como você descreve sua condição de emprego hoje?

☐ Empregado em período integral ‘o dia todo’ (incluir trabalhando como autônomo)

☐ Empregado em meio período (incluir trabalhando como autônomo)

☐ Trabalho casualmente

☐ Não trabalho devido a problemas de saúde

☐ Desempregado

☐ Aposentado

10. Qual foi a faixa de renda da família no último mês?

☐ Até R$998

☐ Entre R$998 e R$1497

☐ Entre R$1498 e R$1996

☐ Maior do que R$1997

11. Você recebe apoio financeiro do governo por meio do ‘Programa Bolsa Família’ por sua criança frequenter a creche/pré-escola?

☐ Sim

☐ Não

12. Quantas pessoas moram na sua casa? (incluindo você).

☐ Um

☐ Dois

☐ Três

☐ Quatro

☐ Cinco ou mais

Sobre a Atividade Física de sua Criança

As perguntas a seguir são sobre a atividade física semanal da sua criança.

Pense um momento sobre um dia de semana normal da sua criança no último mês.

13. Quanto tempo você diria que a sua criança passa brincando ao ar livre em um dia normal da **semana**?

Horas: ☐☐ Minutos: ☐☐

Agora pense um momento sobre um dia de final de semana normal da sua criança no último mês.

14. Quanto tempo você diria que a sua criança passa brincando ao ar livre em um dia normal de **final de semana**?

Horas: ☐☐ Minutos: ☐☐

Sobre o Tempo de Tela da sua Criança

As perguntas a seguir são sobre o tempo de tela semanal da sua criança. Isto inclui o uso de televisão, iPads/tablets, telefones celulares, videogames e computadores.

15. Pensando no último mês, quanto tempo a sua criança gasta fazendo cada uma das seguintes atividades em casa? Por favor, informe separadamente para os dias da semana e para os dias do final de semana.

	Em um dia normal da **semana**<br>Segunda - Sexta<br>horas/minutos	Em um dia normal do **final de semana**<br>Sábado e Domingo<br>horas/minutos
**A. Assistindo TV/DVDs**	☐☐ **hrs** ☐☐ **mins**	☐☐ **hrs** ☐☐ **mins**
**B. Usando o computador**	☐☐ **hrs** ☐☐ **mins**	☐☐ **hrs** ☐☐ **mins**
**C. Jogando videogame (e.g. Nintendo DS, Playstation, Xbox, etc.)**	☐☐ **hrs** ☐☐ **mins**	☐☐ **hrs** ☐☐ **mins**
**D. Usando telefone celular e Ipads/Tablet**	☐☐ **hrs** ☐☐ **mins**	☐☐ **hrs** ☐☐ **mins**

Sobre o Sono da sua Criança

As perguntas a seguir estão relacionadas as rotinas de sono da sua criança.

Pensando no último mês...

16. Que horas a sua criança geralmente dorme a noite?

Em um dia normal da **semana**: Horas: ☐☐ Minutos: ☐☐

Em um dia normal do **final de semana**: Horas: ☐☐ Minutos: ☐☐

17. Que horas a sua criança geralmente acorda de manhã para começar o dia?

Em um dia normal da **semana**: Horas: ☐☐ Minutos: ☐☐

Em um dia normal do **final de semana**: Horas: ☐☐ Minutos: ☐☐

Sobre suas Interações com a sua Criança em Relação a Atividade Física e Tempo de Tela

As perguntas a seguir são sobre como você interage com sua criança em relação a atividade física e tempo de tela. Estamos interessados no que **você** faz e como **você** se sente. Por favor, leia cada questão e selecione a melhor resposta para você. Leve o tempo que precisar e responda com a maior precisão possível.

18. Com que **frequência** a TV da sua casa fica ligada quando tem pessoas em casa?

☐ Muito raramente

☐ Raramente

☐ Às vezes

☐ Frequentemente

☐ Muito frequentemente

☐ Sempre

19. Marque um “X” no quadrado abaixo que melhor representa a frequência com que a sua criança faz cada uma das seguintes atividades enquanto brinca **dentro de sua casa.**

	Sempre	Algumas vezes	Nunca
a. Saltitar, pular, galopar	☐	☐	☐
b. Correr	☐	☐	☐
c. Brincar de pega-pega	☐	☐	☐
d. Brincar se rolando no chão (lutar no chão)	☐	☐	☐
e. Saltar de um lugar mais alto	☐	☐	☐
f. Dar cambalhota (pulando ou caindo)	☐	☐	☐
g. Escalar	☐	☐	☐
h. Se balançar ou ficar pendurado	☐	☐	☐
i. Se equilibrar	☐	☐	☐
j. Juntar travesseiros e pular sobre eles	☐	☐	☐
k. Lançar, jogar ou chutar uma bola	☐	☐	☐

20. Marque um “X” no quadrado abaixo que melhor representa o quanto você concorda ou não concorda com cada uma das seguintes frases.

	Não concordo totalmente	Não concordo	Não concordo e nem discordo	Concordo	Concordo totalmente
a) Quando minha criança está **dentro de casa**, ele/ela deve brincar de forma quieta sem fazer muito barulho.	☐	☐	☐	☐	☐
b) Quando **dentro de casa**, minha criança pode usar brinquedos e equipamentos para jogos fisicamente ativos (*por exemplo, atividades motoras como correr, saltar, pular ou dar cambalhotas*).	☐	☐	☐	☐	☐

21. Com que **frequência** você pede a sua criança para se aquietar quando ele/ela está brincando **dentro de casa**?

☐ Nunca

☐ Muito raramente

☐ Raramente

☐ Algumas vezes

☐ Frequente

☐ Muito frequente

22. Marque um "X" no quadrado abaixo que melhor representa a frequência com que você faz cada uma das seguintes coisas relacionadas ao **jogo ao ar livre** da sua criança.

**Com que frequência você…**	**Nunca**	**Muito raramente**	**Raramente**	**Às vezes**	**Frequente**	**Muito frequente**
a. pede a sua criança para **não** correr quando ele/ela brinca **fora de casa**?	☐	☐	☐	☐	☐	☐
b. pede a sua criança para tentar manter-se limpa quando ele/ela brinca **fora de casa**?	☐	☐	☐	☐	☐	☐
c. deixa a sua criança brincar **fora de casa** em dias quentes?	☐	☐	☐	☐	☐	☐
d. deixa a sua criança brincar **fora de casa** em dias frios?	☐	☐	☐	☐	☐	☐
e. pede a sua criança para se aquietar quando ele/ela brinca **fora de casa**?	☐	☐	☐	☐	☐	☐
f. pede a sua criança para não sujar a roupa quando ele/ela brinca **fora de casa**?	☐	☐	☐	☐	☐	☐

23. Você limita a quantidade de tempo que a sua criança assiste TV, vídeos ou filmes **durante a semana** (segunda a sexta-feira)?

☐ Sim

☐ Não ⟶ **Se "NÃO", pule para a questão 25**

24. Quanto tempo a sua criança tem permissão para assistir a **cada dia da semana**?

Horas: ☐☐ Minutos: ☐☐

25. Você limita a quantidade de tempo que a sua criança assiste TV, vídeos ou filmes **no final de semana** (sábado a domingo)?

☐ Sim

☐ Não ⟶ **Se "NÃO", pule para a questão 27**

26. Quanto tempo a sua criança tem permissão para assistir a **cada dia do final de semana**?

Horas: ☐☐ Minutos: ☐☐

27. Você limita a quantidade de tempo que a sua criança joga videogames **durante a semana** (segunda a sexta-feira)?

☐ Sim

☐ Não ⟶ **Se "NÃO", pule para a questão 29**

28. Quanto tempo a sua criança tem permissão para jogar videogames a **cada dia da semana**?

Horas: ☐☐ Minutos: ☐☐

29. Você limita a quantidade de tempo que a sua criança joga videogames **no final de semana** (sábado a domingo)?

☐ Sim

☐ Não ⟶ **Se "NÃO", pule para a questão 31**

30. Quanto tempo a sua criança tem permissão para jogar videogames a **cada dia do final de semana**?

Horas: ☐☐ Minutos: ☐☐

31. Marque um "X" no quadrado abaixo que melhor representa a frequência com que você faz cada uma das seguintes coisas.

**Com que frequência você…**	**Nunca**	**Muito raramente**	**Raramente**	**Às vezes**	**Frequente**	**Muito frequente**
a. oferece TV, vídeo, ou filme a sua criança como recompensa por um bom comportamento?	☐	☐	☐	☐	☐	☐
b. tira o tempo de TV, vídeo ou filme da sua criança como punição por um mal comportamento?	☐	☐	☐	☐	☐	☐
c. oferece esportes ou atividades físicas a sua criança como recompensa por um bom comportamento?	☐	☐	☐	☐	☐	☐
d. usa esportes ou atividades físicas para fazer com que a sua criança faça algo? (*por exemplo: "você não poderá brincar lá fora até almoçar".*)	☐	☐	☐	☐	☐	☐

32. Marque um "X" no quadrado abaixo que melhor representa o quanto você concorda ou não concorda com cada uma das seguintes frases.

**Eu monitoro de forma rigorosa o tempo que minha criança…**	**Não concordo totalmente**	**Não concordo**	**Não concordo e nem discordo**	**Concordo**	**Concordo totalmente**
a. assiste TV ou vídeos durante a **semana** (segunda a sexta-feira)	☐	☐	☐	☐	☐
b. assiste TV ou vídeos durante o **final de semana** (sábado e domingo)	☐	☐	☐	☐	☐
c. joga videogames durante a **semana** (segunda a sexta-feira)	☐	☐	☐	☐	☐
d. joga videogames durante o **final de semana** (sábado e domingo)	☐	☐	☐	☐	☐

33. Quantos **dias por semana** sua família deixa a televisão ligada durante o **café da manhã**?

☐ 0 dias

☐ 1 dia

☐ 2 dias

☐ 3 dias

☐ 4 dias

☐ 5 dias

☐ 6 dias

☐ 7 dias

34. Quantos **dias por semana** sua família deixa a televisão ligada durante **o jantar**?

☐ 0 dias

☐ 1 dia

☐ 2 dias

☐ 3 dias

☐ 4 dias

☐ 5 dias

☐ 6 dias

☐ 7 dias

35. Marque um "X" no quadrado abaixo que melhor representa a frequência com que você faz cada uma das seguintes coisas.

**Com que frequência…**	**Nunca**	**Muito raramente**	**Raramente**	**Às vezes**	**Frequente**	**Muito frequente**
a. **sua criança** ganha um tempo extra para assistir TV, vídeos ou filmes como recompensa?	☐	☐	☐	☐	☐	☐
b. **sua criança** ganha um tempo extra para brincar fora de casa como recompensa?	☐	☐	☐	☐	☐	☐
c. **você** usa o tempo de TV para controlar o comportamento da sua criança*? (por exemplo: "se você não parar com isto, não poderá assistir TV hoje.")*	☐	☐	☐	☐	☐	☐
d. **você** usa o esporte ou atividades físicas para controlar o comportamento da sua criança*? (por exemplo: "se você não parar com isto, não vai poder ir ao futebol/aula de dança hoje a tarde.")*	☐	☐	☐	☐	☐	☐
e. **você** diminue o tempo da sua criança de brincar do lado de fora de casa por mau comportamento?	☐	☐	☐	☐	☐	☐

36. Marque um "X" no quadrado abaixo que melhor representa o quanto você concorda ou não concorda com cada uma das seguintes frases.

	Não concordo totalmente	Não concordo	Não concordo e nem discordo	Concordo	Concordo totalmente
a) **Eu** tenho controle sobre o quanto de tempo de TV minha criança assiste.	☐	☐	☐	☐	☐
b) **Outros adultos** que convivem na vida da minha criança dificultam que ele/ela seja fisicamente ativo(a).	☐	☐	☐	☐	☐
c) Minha **família** é fisicamente ativa.	☐	☐	☐	☐	☐
d) **Eu** gosto de assistir TV/filmes com minha criança.	☐	☐	☐	☐	☐

37. O quanto **você** gosta de atividade física ou esporte?

☐ Não gosto

☐ Gosto um pouco

☐ Gosto razoavelmente

☐ Gosto muito

38. O quanto **você** gosta de assistir TV ou filmes durante o seu tempo livre?

☐ Não gosto

☐ Gosto um pouco

☐ Gosto razoavelmente

☐ Gosto muito

39. Com que frequência **sua família** usa a prática de atividades físicas ou esportes como uma forma de recreação familiar *(por exemplo, andar de bicicleta juntos, caminhar, jogar futebol/vôlei)?*

☐ Raramente

☐ De vez em quando

☐ Com relativa frequência

☐ Frequentemente

40. Com que frequência **você** vai assistir a **sua criança** em eventos esportivos, aulas ou competições organizadas de atividades físicas com ele/ela *(por exemplo, assistir a sua criança em competição de futebol ou vôlei, apresentação de dança, ou sessões de treinamento)*?

☐ Raramente

☐ De vez em quando

☐ Com relativa frequência

☐ Frequentemente

41. Quão importante é para **você** que a **sua criança** seja fisicamente ativa?

☐ Não é importante

☐ De pouco valor

☐ Moderadamente importante

☐ Importante

☐ Muito importante

42. Durante o **ano passado**, algum adulto da sua família **pagou** para que a sua criança pudesse ter aulas ou praticar esportes que envolvessem atividade física moderada ou vigorosa *(por exemplo, dança, futebol, basquete, natação, ginástica)?*

☐ Sim

☐ Não → **Se "NÃO", pule para a questão 44**

43. Para quantas atividades você ou outro adulto pagou?

☐☐ **Atividades**

44. Com que frequência você usa **seu próprio comportamento** para incentivar a sua criança a ser fisicamente ativa?

☐ Eu não uso meu próprio comportamento para incentivar minha criança a ser ativa.

☐ Eu raramente uso meu próprio comportamento para incentivar minha criança a ser ativa.

☐ Eu frequentemente uso meu próprio comportamento para incentivar minha criança a ser ativa.

☐ Eu sempre uso meu próprio comportamento para incentivar minha criança a ser ativa.

45. Com que frequência você matricula a sua criança em esportes?

☐ Eu raramente matriculo minha criança em esportes.

☐ De vez em quando eu matriculo minha criança em esportes.

☐ Eu frequentemente matriculo minha criança em esportes.

☐ Eu sempre matriculo minha criança em esportes.

46. Durante o **último mês**, quantas vezes **você** levou a sua criança para brincar em um parque, praça, ou outra área de lazer da região?

☐☐ **Vez(es) no último mês**

47. Marque um "X" no quadrado abaixo que melhor representa o quanto você concorda ou não concorda com cada uma das seguintes frases.

	**Não concordo totalmente**	**Não concordo**	**Não concordo e nem discordo**	**Concordo**	**Concordo totalmente**
a. **Eu** sou o responsável pela quantidade de TV que minha criança assiste durante seu tempo livre em casa.	☐	☐	☐	☐	☐
b. **Eu** posso fazer com que minha criança seja fisicamente ativa em casa.	☐	☐	☐	☐	☐
c. **Outros adultos** que tem contato com a minha criança dificultam as regras de casa sobre o tempo permitido para ele/ela assistir televisão.	☐	☐	☐	☐	☐
d. **Eu** gosto de ser fisicamente ativo(a) com minha criança.	☐	☐	☐	☐	☐

48. Marque um "X" no quadrado abaixo que melhor representa a frequência com que você faz cada uma das seguintes coisas durante uma semana normal.

**Durante uma semana normal, com que frequência...**	**Nunca**	**Muito raramente**	**Raramente**	**Algumas vezes**	**Frequente**	**Muito frequente**
a. **você** diz a sua criança como os hábitos sedentários *(por exemplo, ficar sentado por muito tempo)* podem ser prejudiciais para a saúde?	☐	☐	☐	☐	☐	☐

b. **você** assiste TV ou vídeos com a sua criança?	☐	☐	☐	☐	☐	☐
c. **você** manda a sua criança ir brincar fora de casa para que você possa fazer as atividades domésticas da sua casa?	☐	☐	☐	☐	☐	☐
d. **você** leva a sua criança para brincar no parque?	☐	☐	☐	☐	☐	☐

49. Marque um "X" no quadrado abaixo que melhor representa a frequência com que cada uma das seguintes coisas acontecem durante uma semana normal.

**Durante uma semana normal, com que frequência...**	**Nunca**	**Muito raramente**	**Raramente**	**Algumas vezes**	**Frequente**	**Muito frequente**
a. **você** diz a sua criança que fazer atividade física é bom para a saúde?	☐	☐	☐	☐	☐	☐
b. **seu** comportamento incentiva sua criança a ser sedentária? *(por exemplo, assistir TV por muito tempo.)*	☐	☐	☐	☐	☐	☐
c. **você** elogia a sua criança por participar em esportes ou atividades físicas?	☐	☐	☐	☐	☐	☐
d. **você** liga a TV, um vídeo ou um filme para sua criança assistir quando o tempo está ruim? *(por exemplo, quando está chovendo, muito quente ou muito frio.)*	☐	☐	☐	☐	☐	☐
e. **você** diz coisas para incentivar a sua criança a fazer atividades físicas ou praticar esportes?	☐	☐	☐	☐	☐	☐

50. Marque um "X" no quadrado abaixo que melhor representa a frequência com que cada uma das seguintes coisas acontecem durante uma semana normal.

**Durante uma semana normal, com que frequência...**	**Nunca**	**Muito raramente**	**Raramente**	**Algumas vezes**	**Frequente**	**Muito frequente**
a. a sua criança **ouve você dizer** que estava cansado(a) demais para fazer atividades físicas?	☐	☐	☐	☐	☐	☐
b. a sua criança **vê** você assistindo TV ou filmes?	☐	☐	☐	☐	☐	☐

c. **você** pratica esportes, participa de jogos ativos ou faz outras atividades físicas com a sua criança?	☐	☐	☐	☐	☐	☐
d. **você** tenta incentivar a sua criança a brincar fora de casa quando o tempo está bom?	☐	☐	☐	☐	☐	☐
e. **você** leva a sua criança a algum lugar onde ele/ela possa ser fisicamente ativo(a) ou praticar esportes?	☐	☐	☐	☐	☐	☐

51. Marque um "X" no quadrado abaixo que melhor representa a frequência com que cada uma das seguintes coisas acontecem durante uma semana normal.

**Durante uma semana normal, com que frequência...**	**Nunca**	**Muito raramente**	**Raramente**	**Algumas vezes**	**Frequente**	**Muito frequente**
a. a sua criança **ouve você** falar sobre participar de algum esporte ou ser fisicamente ativo(a)?	☐	☐	☐	☐	☐	☐
b. a sua criança **vê** você fazendo ou indo fazer algo que seja fisicamente ativo *(por exemplo, caminhar, andar de bicicleta, praticar esportes)?*	☐	☐	☐	☐	☐	☐
c. **você** liga a TV, um vídeo ou um filme para sua criança para que você possa fazer as atividades domésticas da sua casa?	☐	☐	☐	☐	☐	☐
d. **você** tenta fazer com que a sua criança seja fisicamente ativa ao invés de assistir TV?	☐	☐	☐	☐	☐	☐
e. **você** diz coisas para incentivar a sua criança a gastar menos tempo sendo sedentário*? (por exemplo, "pare de assistir e vá para rua brincar.")*	☐	☐	☐	☐	☐	☐

52. Marque um "X" no quadrado abaixo que melhor representa o quão importante cada uma das seguintes frases é para você.

**O quão importante é para você que a sua criança...**	**Não é importante**	**Um pouco importante**	**Moderadamente importante**	**Importante**	**Muito importante**
a. participe em esportes?	☐	☐	☐	☐	☐
b. seja fisicamente ativa quando crescer?	☐	☐	☐	☐	☐

Sobre suas Interações com a sua Criança em Relação ao Sono

As perguntas a seguir são sobre como você interage com a sua criança em relação ao sono.

Para responder as questões lembre-se que:
As rotinas de dormir são uma sequência definida de atividades que ocorrem geralmente na mesma ordem e com o mesmo adulto responsável pela criança antes dele/dela dormir. Marque um "X" no quadrado abaixo que melhor representa a rotina de dormir da sua criança no último mês.

**53. Durante as noites da *semana* do último mês, com que frequência a sua criança…**	**Quase nunca**	**Algumas vezes**	**Metade das vezes**	**Frequente**	**Quase sempre**
a) realizou as **mesmas atividades** na hora antes de ir para a cama *(por exemplo, tomar banho, escovar os dentes, ler ou ouvir histórias, ouvir música)*?	☐	☐	☐	☐	☐
b) realizou as atividades **na mesma ordem** antes de ir para a cama *(por exemplo, tomar banho, escovar os dentes, ler ou ouvir histórias, ouvir música)*?	☐	☐	☐	☐	☐
c) dormiu **no mesmo lugar** *(por exemplo, na cama dele/dela, na cama dos pais, no sofá)*?	☐	☐	☐	☐	☐
d) foi para a cama **no mesmo horário** (dentro de 10 minutos do horário previsto)?	☐	☐	☐	☐	☐
e) foi colocado para dormir **pelo mesmo adulto responsável**?	☐	☐	☐	☐	☐

**54. Durante as noites do *final de semana* do último mês, com que frequência a sua criança...**	**Quase nunca**	**Algumas vezes**	**Metade das vezes**	**Frequente**	**Quase sempre**
a) realizou as **mesmas atividades** na hora antes de ir para a cama *(por exemplo, tomar banho, escovar os dentes, ler ou ouvir histórias, ouvir música)*?	☐	☐	☐	☐	☐
b) realizou as atividades **na mesma ordem** antes de ir para a cama *(por exemplo, tomar banho, escovar os dentes, ler ou ouvir histórias, ouvir música)*?	☐	☐	☐	☐	☐
c) dormiu **no mesmo lugar** *(por exemplo, na cama dele/dela, na cama dos pais, no sofá)*?	☐	☐	☐	☐	☐
d) foi para a cama **no mesmo horário** (dentro de 10 minutos do horário previsto)?	☐	☐	☐	☐	☐
e) foi colocado para dormir **pelo mesmo adulto responsável**?	☐	☐	☐	☐	☐

55. Quão chateado a sua criança fica se ele/ela NÃO…	Nenhum pouco	Um pouco	Moderada-mente	Bastante	Extrema-mente
f) realiza as **mesmas atividades** na hora antes de ir para a cama (*por exemplo, tomar banho, escovar os dentes, ler ou ouvir histórias, ouvir música*)?	☐	☐	☐	☐	☐
g) realiza as atividades **na mesma ordem** antes de ir para a cama (*por exemplo, tomar banho, escovar os dentes, ler ou ouvir histórias, ouvir música*)?	☐	☐	☐	☐	☐
h) dorme **no mesmo lugar** (*por exemplo, na cama dele/dela, na cama dos pais, no sofá*)?	☐	☐	☐	☐	☐
i) vai para a cama **no mesmo horário** (dentro de 10 minutos do horário previsto)?	☐	☐	☐	☐	☐
j) for colocado para dormir **pelo mesmo adulto responsável**?	☐	☐	☐	☐	☐

56. No mês passado, na hora antes de ir para cama, com que frequência a sua criança…	Quase nunca	Algumas vezes	A metade das vezes	Freque nte	Quase sempre
a. Lêu/ouviu uma história?	☐	☐	☐	☐	☐
b. Brincou com jogos ou brinquedos?	☐	☐	☐	☐	☐
c. Brincou de forma ativa *(por exemplo, correu ou deu cambalhota)*?	☐	☐	☐	☐	☐
d. Assitiu TV?	☐	☐	☐	☐	☐
e. Jogou videogame?	☐	☐	☐	☐	☐
f. Escutou música?	☐	☐	☐	☐	☐
g. Comeu um lanche ou bebeu algo?	☐	☐	☐	☐	☐
h. Tomou um banho?	☐	☐	☐	☐	☐
i. Escovou os dentes?	☐	☐	☐	☐	☐
j. Usou o banheiro (privada)?	☐	☐	☐	☐	☐
k. Abraçou/beijou o adulto responsável (*por exemplo, deu um beijo de boa noite*)?	☐	☐	☐	☐	☐
l. Disse boa noite aos membros da família?	☐	☐	☐	☐	☐

m.	Foi colocado na cama?	☐	☐	☐	☐	☐
n.	Colocou o pijama/roupa de dormir?	☐	☐	☐	☐	☐
o.	Fez orações?	☐	☐	☐	☐	☐
p.	Abraçou o adulto responsável *(por exemplo, a criança sentou no colo do responsável e ficou abraçado com ele/ela)*?	☐	☐	☐	☐	☐

Você terminou!

Muito obrigada pelo seu tempo e esforço!
